# Evaluation of the Effects on Uninfected Pregnant Women and Their Pregnancy Outcomes During the COVID-19 Pandemic in Beijing, China

**DOI:** 10.3389/fmed.2022.842826

**Published:** 2022-05-11

**Authors:** Chongyi Hao, Feng Jin, Chanjuan Hao, Xiaofen Zhang, Limin Xie, Yawei Zhang, Xuanshi Liu, Xin Ni, Wei Li

**Affiliations:** ^1^Beijing Key Laboratory for Genetics of Birth Defects, Beijing Pediatric Research Institute, Beijing, China; ^2^MOE Key Laboratory of Major Diseases in Children, Beijing, China; ^3^Rare Disease Center, National Center for Children's Health, Beijing, China; ^4^Beijing Children's Hospital, Capital Medical University, Beijing, China; ^5^Shunyi Women's and Children's Hospital of Beijing Children's Hospital, Beijing, China; ^6^Department of Cancer Prevention and Control, National Cancer Center/National Clinical Research Center for Cancer/ Cancer Hospital, Chinese Academy of Medical Sciences and Peking Union Medical College, Beijing, China; ^7^National Center for Children's Health, Beijing, China

**Keywords:** COVID-19, adverse pregnancy outcomes, birth cohort, lifestyle, nested case-control study

## Abstract

**Background:**

People's lifestyles may have changed during the COVID-19 pandemic, which may have a profound impact on pregnant women and newborns. This study aims to assess the effects of the COVID-19 pandemic on uninfected pregnant women and their newborns, including potential environmental factors.

**Methods:**

We retrospectively analyzed the pregnancy complications of 802 cases in the pandemic group and 802 controls in the pre-pandemic group in a matched nested case-control study, and evaluated the association with sociodemographic features, lifestyles, and other factors in 311 pregnant women with adverse pregnancy outcomes.

**Results:**

Compared to the pre-pandemic group, the rates of anemia, vaginitis, shoulder dystocia, and adverse pregnancy outcomes such as preterm birth were increased in the pandemic group. After controlling for the covariates, we observed a higher risk of adverse pregnancy outcomes in the pandemic group. Pregnant women with adverse pregnancy outcomes had an increased rate of anemia and vaginal candidiasis.

**Conclusion:**

COVID-19 pandemic has profound effects on adverse pregnancy outcomes, suggesting the importance of ensuring regular prenatal checkups and keeping a healthy lifestyle.

## Introduction

The outbreak of COVID-19 has a profound impact on global healthcare systems, social structures, and the world economy (1–3). To prevent the spread of COVID-19, many governments have implemented regional blockades and restrictions on free activities (4–7). These regulations, together with the reduction of routine medical visits (5, 8) and the panic during the pandemic (9), may change the life behaviors of pregnant women (10, 11), thus affecting the outcomes of pregnancy (12). To evaluate the effects, most studies have focused on the pregnancy outcomes of women infected with COVID-19, such as cesarean section rate (13, 14), postpartum complications (14), and preterm birth (13). However, uninfected pregnant women account for a much larger population in countries or regions with lower rate of SARS-CoV-2 infection, and few results on maternal and fetal outcomes during the COVID-19 pandemic were reported with inconsistent results. Some studies found a higher rate of adverse pregnancy outcomes in pregnant women during the COVID-19 pandemic, such as gestational hypertension (15), gestational diabetes (15), premature rupture of membranes (16), stillbirth (1, 17), and preterm birth (18). However, Chmielewska et al. (1) found that preterm birth in high-income countries decreased during the COVID-19 pandemic, but Du et al. (16) and Handley et al. (19) did not find a change in preterm births during the pandemic. Moreover, studies rarely focused on the impact of environmental factors on adverse pregnancy outcomes. Therefore, we aim to investigate the adverse pregnancy outcomes of uninfected pregnant women during pandemic in Shunyi, Beijing, and to explore the medical, social, environmental factors in relation to adverse pregnancy outcomes.

A matched nested case-control study was performed using the China Longitudinal Environmental, Genetic, and Economic Cohort (CHALLENGE) from Shunyi Maternal and Children's Hospital of Beijing Children's Hospital. We compared clinical diagnoses during pregnancy and at labor between the pandemic group (delivery time from April to September 2020) and the pre-pandemic group (delivery time from April to September 2019). We further explored the environmental factors in relation to adverse pregnancy outcomes to provide evidence guiding prenatal care during the COVID-19 pandemic and long-term social isolation.

## Materials and Methods

### Study Subjects and Information Collection

**Figure 1:**
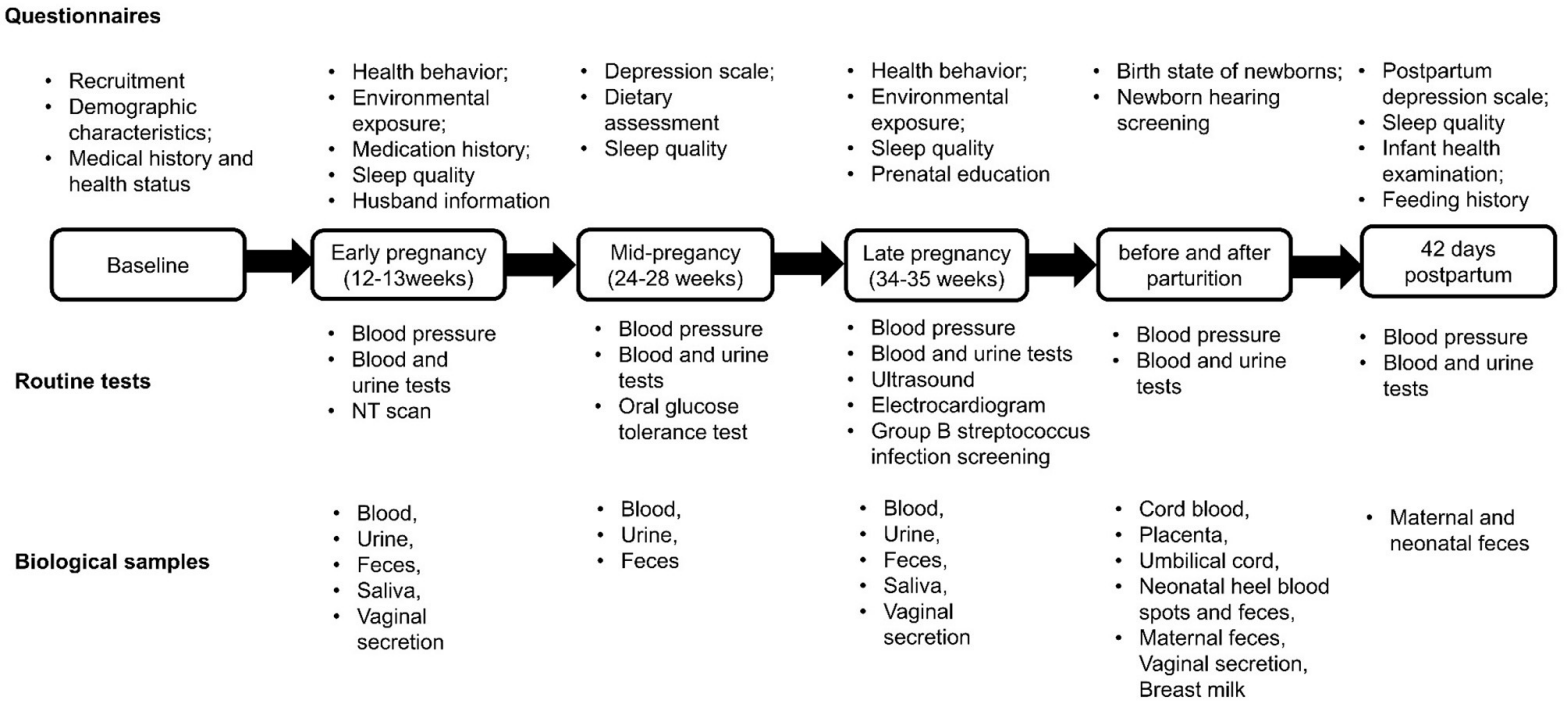
Questionnaires, routine tests and biological samples were collected at different time points in the CHALLENGE birth cohort. Six types of questionnaires including baseline information, environmental exposures, physical exercise sleep quality, depression scale, and dietary information were collected. After the baseline information was collected, routine tests were conducted in 5 follow-up visits including three routine prenatal testing at 12–13 weeks, 24–28 weeks and 34–35 weeks, one postpartum checkup, and one postnatal checkup at 42 days after parturition. During these visits, blood pressure, blood tests, urine tests and additional tests such as group B streptococcus infection screening, ultrasound exam, nuchal translucency (NT) scan, and electrocardiogram were conducted. The biological samples were collected as shown at different time points.

This matched nested case-control study included pregnant women and their newborns from the CHALLENGE study conducted at Shunyi Maternal and Children's Hospital of Beijing Children's Hospital between March 2018 and April 2021. Criteria for inclusion were pregnant women with (1) age ≥ 18 years, (2) gestational weeks ≤ 13 weeks, (3) having lived in the Shunyi area for at least 1 year without moving plan in recent 5 years, and (4) no diagnosed mental illness or intellectual disability. In the CHALLENGE study, all women were recruited at their first trimester of pregnancy and conducted 5 follow-up visits during their pregnancy and after their delivery. Eligible pregnant women were asked to sign the informed consent forms and completed baseline information. Five follow-up visits included three routine prenatal tests at 12–13 weeks, 24–28 weeks and 34–35 weeks, one postpartum checkup, and one postnatal checkup at 42 days after parturition. During these visits, routine tests including blood pressure, blood tests, and urine tests were conducted. Additional tests were performed at specific time points, such as group B streptococcus infection screening, ultrasound exam, nuchal translucency (NT) scan, and electrocardiogram (Figure 1). Along with each routine prenatal testing, online questionnaires were applied to collect baseline information, environmental exposures, sleep quality, depression scale, and dietary information. We constructed a database to integrate electronic medical records from hospital information systems and routine laboratory tests. The cohort had recruited 9,635 pregnant women and 8,183 babies as of April 2021. All data had been safely stored and deidentified. The chronological order of questionnaires, routine tests, and biological samples of CHALLENGE study are shown in Figure 1.

The pregnant women completed a baseline questionnaire at the time of recruitment, including demographic characteristics (age, ethnicity, educational level, address, institution of employment) of pregnant women and their spouses, health survey (including pre-pregnancy body weight and height, date of last menstrual period, basic information, development history, and physical examination, birth history, medication history for diseases) of woman. At 12 to 13 weeks of gestation, the questionnaire focused on life behaviors (such as smoking, alcohol drinking, tea drinking, nutrient supplements, physical activities, and use of electronic products), environmental exposures (work and residential environment), medication history during pregnancy and spouse's life behaviors. The above mentioned two questionnaires are shown in the Supplementary Material.

### Study Design

**Figure 2:**
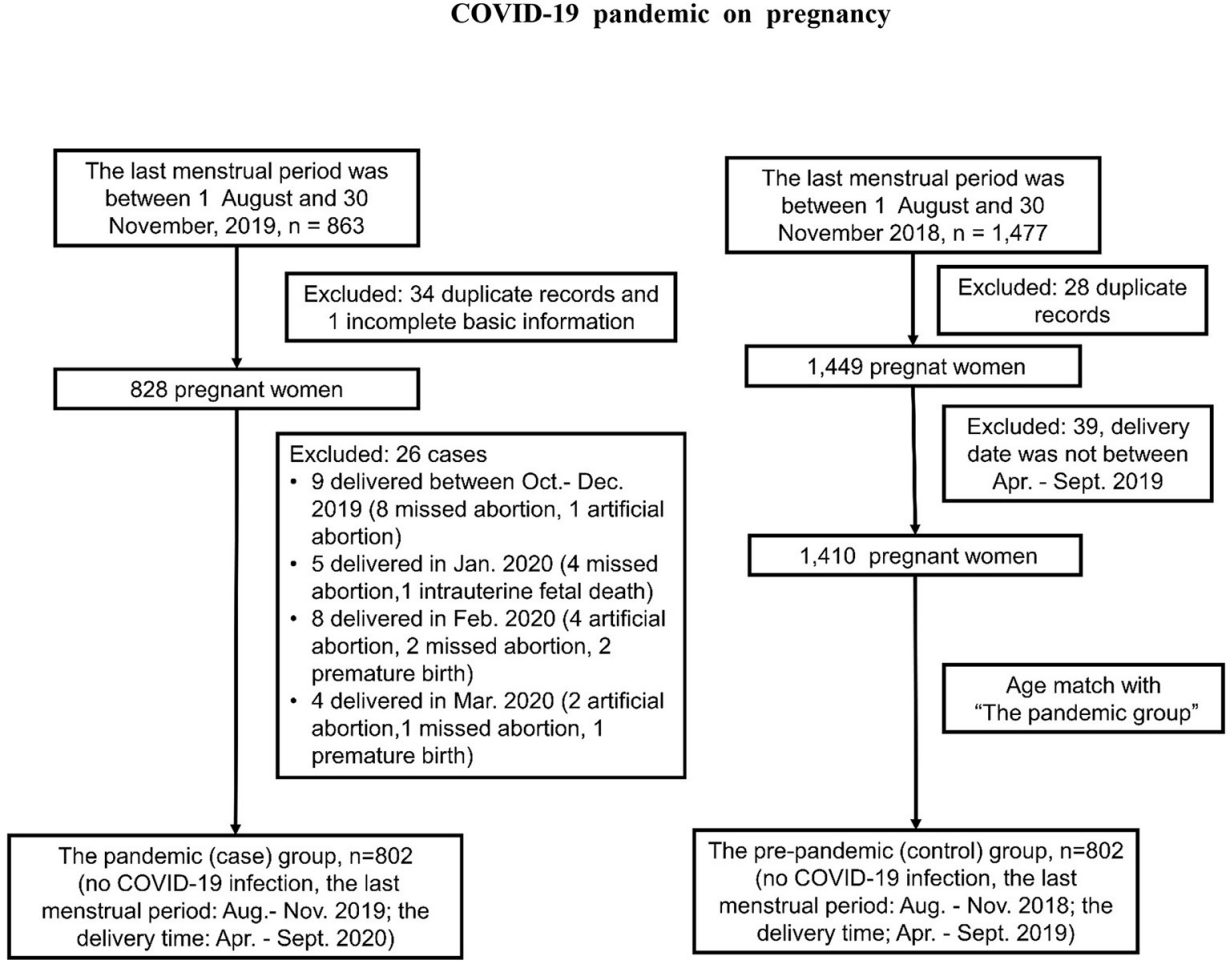
A flowchart of participant selection and exclusion in this study, China, 2018-2020.

To explore the impact of COVID-19 pandemic on uninfected pregnant women, we conducted a nested case-control study building upon the CHANLLEGE study. Based on the estimated incidence rate of adverse pregnancy outcomes (0.4–0.9%) in northern China, the sample size was calculated as at least 562 cases and 562 controls to reach a power of 80% and a type I error of 5%. We divided the study objects into pandemic (case) and pre-pandemic (control) group from the CHALLENGE study, and none of them were infected with COVID-19. For the pandemic group, we chose pregnant women whose last menstrual period were from August 1, 2019 to November 30, 2019. 802 out of 863 participants of CHANLLEGE cohort were recruited in the pandemic group. These pregnant women delivered from April to September, 2020 during COVID-19 pandemic when strict measures of social isolation was executed in Beijing. As for the pre-pandemic group in matched period, we selected pregnant women who had their last menstruation between 1 August, 2018 and 30 November, 2018. They all delivered their babies before the COVID-19 pandemic. After matching the maternal age and delivery time with the pandemic group, 802 pregnant women out of 1,477 participants of CHANLLEGE study were included. The detailed selection criteria were shown in Figure 2.

### Variables and Adverse Pregnancy Outcomes

All relevant diagnoses were obtained from the medical records, including pregnancy-related maternal diagnoses (anemia, vaginitis, infection), pregnancy-specific disorder (gestational diabetes mellitus, gestational hypertension, preeclampsia), pregnancy complications (placental and fetal membrane abnormalities, umbilical cord abnormalities), delivery complications (postpartum hemorrhage, laceration of cervix during labor, perineal laceration during delivery unspecified, shoulder dystocia), and adverse pregnancy outcomes (preterm birth, macrosomia, large for gestational age, small for gestational age, fetal death, low weight infants, congenital abnormalities). Based on corresponding diagnostic guidelines, the adverse pregnancy outcomes were defined as following. Preterm birth was defined as a birth before 37 weeks of gestation. Macrosomia was defined as a newborn whose birth weight was more than 4,000 g and low birth weight was defined as <2,500 g. Large for gestational age (LGA) referred to an infant whose birth weight was above the 90th percentile of the average weight for the same gestational age. Small for gestational age (SGA) referred to an infant whose birth weight fell below the 10th percentile of the average weight for the same gestational age. Stillbirth was defined as a baby born with no signs of life at a gestational age of 20 weeks or more. Congenital abnormalities were defined as structural or functional anomalies that present at birth.

Related environmental factors such as alcohol drinking, tea drinking, smoking, nutrient supplement, and frequency of using computers and watching TV were collected from the standardized questionnaires at early pregnancy (12–13 weeks).

### Statistical Analyses

All statistical analyses were conducted using IBM SPSS Statistics for Windows, Version 25.0 (IBM Corp., Armonk, NY, USA). Qualitative variables were expressed by frequency, and quantitative variables were expressed by mean (x) ± standard deviation (SD). We applied the *X*^2^ test and Mann-Whitney U test to compare the basic characteristics and the pregnancy complications between two groups. The binary logistic regression model was used to investigate the relationship between the COVID-19 pandemic and pregnancy complications after controlling for pregnant women's and their spouse's demographic characteristics (age, ethnicity, education level, work), pre-pregnancy body mass index (BMI), number of pregnancies, bad obstetric history, and history of cesarean section. We performed *X*^2^ test to analyze the factors associated with the adverse pregnancy outcomes that related to the COVID-19 pandemic, and logistic regression model was used for prediction analysis. The statistical significance level has been set to *P* = 0.05.

### Ethical Considerations

All study procedures were approved by the Institutional Review Board (IRB) at the Shunyi Maternal and Children's Hospital of Beijing Children's Hospital with the approval IRB number of 2021-01. Informed written consents were obtained from all participants at the initial hospital visits.

## Results

### Participant Characteristics and Rates of Stay-at-Home During COVID-19 Pandemic

A total of 1,604 pregnant women (mean age, 32.48 ± 3.85 years) were enrolled in this study including age-matched 802 participants (mean age, 32.40 ± 3.82 years) in the pandemic group and 802 participants (mean age, 32.56 ± 3.88 years) in the pre-pandemic group, respectively (Figure 2). Among 1,604 women, 93.52% were Han ethnicity, 47.01% had a bachelor's degree or above, 66.96% of pregnant women received pregnancy education at their initial visit to the hospital, and their pre-pregnancy BMI was 22.61 ± 3.79 kg/m^2^. The average age of the spouse was 33.65 ± 4.41 years old, in whom 93.81% were Han ethnicity, and 43.62% had a bachelor's degree or above. We found that the rates of stay-at-home were higher in the pandemic group (pregnant women: 16.3 vs. 13%; spouses: 17.5 vs. 11.5%), especially in the spouse group (*P* = 0.01), while the number of primiparas was significantly higher in the pre-pandemic group (Table 1).

**Table 1:** Characteristics of 1,604 pregnant women before and during the COVID-19 pandemic, China, 2018-2020.

	**Total (*n* = 1,604)**	**Pandemic group (*n* = 802)**	**%**	**Pre-pandemic group (*n* = 802)**	**%**	**X^2^/Z**	***P*-value**
**Maternal age (years)**	32.48 ± 3.85	32.40 ± 3.82		32.56 ± 3.88		−0.355	0.722
**Maternal ethnicity**						0.837	0.658
Han	1,500	746	93.0	754	94.0		
Man	50	28	3.5	22	2.7		
Other	54	28	3.5	26	3.2		
**Maternal education level**						0.36	0.548
Junior college degree or below	850	419	52.2	431	53.7		
Bachelor degree or above	754	383	47.8	371	46.3		
**Pre-pregnancy education**						0.012	0.914
yes	1,066	532	67.1	534	66.8		
no	526	261	32.9	265	33.2		
**Maternal occupation**						3.442	0.064
Employed	1,360	669	83.4	691	86.2		
Stay-at-home	235	131	16.3	104	13.0		
**Maternal height (cm)**	161.79 ± 5.04	161.71 ± 5.00		161.86 ± 5.08		−0.445	0.656
**Pre-pregnancy weight (kg)**	59.20 ± 10.41	59.19 ± 10.28		59.22 ± 10.54		−0.117	0.907
**Pre-pregnancy BMI (kg/m2)**	22.61 ± 3.79	22.63 ± 3.78		22.59 ± 3.80		−0.31	0.756
Underweight <18.5	124	52	6.6	72	9.0		
Normal 18.5–24.9	1,107	566	71.4	541	67.9		
Overweight 25–29.9	290	142	17.9	148	18.6		
Obese ≥ 30	69	33	4.2	36	4.5		
**Primipara ^a^**						5.774	0.016^*^
yes	498	226	28.5	272	34.1		
no	1,091	566	71.5	525	65.9		
**Spouse age (year)**	33.65 ± 4.41	33.50 ± 4.24		33.81 ± 4.58		−0.52	0.603
**Spouse ethnicity**						0.518	0.772
Han	1,500	750	94.0	750	93.6		
Man	59	27	3.4	32	4.0		
Other	40	21	2.6	19	2.4		
**Spouse education**						0.248	0.618
Junior college degree or below	901	445	55.8	456	57.0		
Bachelor degree or above	697	353	44.2	344	43.0		
**Spouse occupation**						10.946	0.010^*^
Employed	1,316	636	79.3	680	84.8		
Stay-at-home	233	140	17.5	93	11.6		

* *P < 0.05*.

a *: Gravidity not included. Missing data: pre-pregnancy education, 12 (0.75%); maternal occupation 9 (0.5%); maternal height, 12 (0.75%); pre-pregnancy weight, 14 (0.87%); pre-pregnancy BMI 14 (0.87%); gravidity 15 (0.94%); spouse age 6 (0.37%); spouse ethnicity 6 (0.37%); spouse education 6 (0.37%); spouse occupation 55 (3.4%)*.

### Comparison of the Pregnancy Complications Between Two Groups

In the primary analysis, we compared the diagnosed complications between two groups during pregnancy and at labor. During pregnancy, the proportion of anemia and vaginitis in the pandemic group was higher than the pre-pandemic group (46.63 vs. 37.16%, *P* < 0.001; 7.11 vs. 2.37%, *P* < 0.001) (Table 2). At labor, the rate of shoulder dystocia was higher in the pandemic group (3.87% vs. 0.75%, *P* < 0.001), but postpartum hemorrhage decreased (21.1 vs. 26.6%, *P* < 0.010) (Table 2). We further assessed the adverse pregnancy outcomes including preterm birth, macrosomia, large for gestational age, small for gestational age, fetal death, low weight infants, and congenital anomalies. We selected 1,604 pregnant women in total. Of them, we found 311 pregnant women who were diagnosed with at least one type of adverse pregnancy outcomes. The overall incidence of adverse pregnancy outcomes was higher in the pandemic group than the pre-pandemic group (21.45 vs. 17.33%, *P* = 0.037), such as preterm birth (5.86 vs. 3.24%, *P* = 0.012) (Table 2).

**Table 2:** Pregnancy outcomes before and during the COVID-19 pandemic, China, 2018-2020.

			**Pandemic group (*n* = 802)**	**%**	**Pre-pandemic group (*n* = 802)**	**%**	**X^**2**^/Z**	***P-*value**
**During pregnancy**
**Maternal related diagnoses**
		Anemia	374	46.6	298	37.2	15.178	<0.001^***^
		Vaginitis^a^	57	7.1	19	2.400	21.829	<0.001^***^
		Infection^b^	97	12.1	117	14.6	2.192	0.139
**Pregnancy-specific disorder**
		Gestational diabetes mellitus	93	11.6	114	14.200	2.446	0.118
		Gestational hypertension	16	2.0	28	3.500	3.370	0.070
		Preeclampsia	27	3.4	23	2.900	0.330	0.565
**Pregnancy complications**
		Placental and fetal membrane abnormalities^c^	219	27.3	213	26.600	0.155	0.694
		Umbilical cord abnormalities^d^	198	24.7	226	28.200	2.513	0.113
**Delivery term**
**Fetal related diagnoses**
		Adverse pregnancy outcomes^e^	172	21.4	139	17.3	4.344	0.037^*^
		Preterm birth	47	5.9	26	3.2	6.329	0.012^*^
		Macrosomia	81	10.1	73	9.1	0.460	0.498
		Large for gestational age	70	8.7	51	6.4	3.227	0.072
		Small for gestational age	27	3.4	25	3.1	0.080	0.778
		Fetal death	1	0.1	2	0.2	—	—
		Low weight infants	1	0.1	5	0.6	—	—
		Congenital anomalies	20	2.5	17	2.1	0.249	0.618
		Fetal distress	72	9.0	70	8.7	0.031	0.86
**Delivery complications**
		Postpartum hemorrhage	169	21.1	213	26.6	6.652	0.010^*^
		Laceration of cervix during labor	12	1.5	22	2.7	3.005	0.083
		Perineal laceration during delivery unspecified	322	40.1	360	44.9	3.683	0.055
		Shoulder dystocia	31	3.9	6	0.7	17.291	<0.001^***^

* *P < 0.05*;

*** *P < 0.001*.

a *: vaginal candidiasis (pandemic group n = 47, pre-pandemic group n = 12), mycoplasma vaginitis (5 vs. 6), others (5 vs. 1)*.

b *: Group B streptococcal infection, mycoplasma infection, intrauterine infection, chorioamnionitis and others*.

c *: Placenta previa (pandemic group n = 5, pre-pandemic group n = 2), Placental abruption (10:10), Premature rupture of membranes (208:206)*.

d *: Umbilical cord abnormalities include cord winding, cord torsion, cord true knot, etc*.

e *: Preterm birth is defined as <37 weeks of gestational age at delivery. Macrosomia refers to newborns weighing more than 4,000 g. Large for gestational age (LGA) refers to an infant whose birth weight is above the 90th percentile of the average weight for the same gestational age. Small for gestational age (SGA) refers to an infant whose birth weight falls below the 10th percentile of the average weight for the same gestational age. Low weight infants refer to newborns weighing <2,500 g*.

We applied several models to control for the confounding factors (Table 3). In the univariate model I, we observed anemia, vaginitis, shoulder dystocia, postpartum hemorrhage, and adverse pregnancy outcomes that were significantly associated with the pandemic. After adjustment for the demographic characteristics (age, ethnicity, education, and work) of pregnant women in model II, all the above pregnancy outcomes remained statistically significant. Apart from the demographic characteristics of pregnant women, we further controlled for the demographic characteristics of their spouses in model III, we observed a higher risk of all above pregnancy outcomes. In model IV, we additionally controlled for pre-pregnancy BMI, gravidity, history of abnormal pregnancy, history of cesarean section, all results remained significant (anemia: OR = 1.498, 95% CI = 1.216–1.846; vaginitis: OR = 3.294, 95% CI = 1.878–5.777; adverse pregnancy outcomes: OR = 1.301, 95% CI = 0.999–1.695; postpartum hemorrhage: OR = 0.779, 95% CI = 0.612–0.992; shoulder dystocia: OR = 5.117, 95% CI = 2.098–12.48; preterm birth: OR = 1.944, 95% CI = 1.144–3.302).

**Table 3:** The influence of the COVID-19 pandemic on pregnancy outcomes, China, 2018-2020.

**Pregnancy outcomes**	**Model I**			**Model II**			**Model III**			**Model IV**		
	***P*-value**	**OR**	**95% CI**	***P-*value**	**OR**	**95% CI**	***P*-value**	**OR**	**95% CI**	***P*-value**	**OR**	**95% CI**
Anemia	<0.001	1.478	(1.211–1.804)	<0.001	1.474	(1.200–1.792)	<0.001	1.445	(1.176–1.775)	<0.001	1.498	(1.216–1.846)
Vaginitis	<0.001	3.153	(1.858–5.350)	<0.001	3.153	(1.856–5.356)	<0.001	3.311	(1.895–5.785)	<0.001	3.294	(1.878–5.777)
Adverse pregnancy outcomes	0.037	1.302	(1.015–1.670)	0.035	1.310	(1.019–1.683)	0.046	1.300	(1.005–1.682)	0.051^a^	1.301	(0.999–1.695)
Postpartum hemorrhage	0.018	0.756	(0.600–0.953)	0.020	0.758	(0.600~0.957)	0.031	0.769	(0.606–0.977)	0.043	0.779	(0.612–0.992)
Shoulder dystocia	<0.001	5.334	(2.213–12.857)	<0.001	5.091	(2.104–12.319)	<0.001	5.250	(2.165–12.732)	<0.001	5.117	(2.098–12.48)
Preterm birth	0.013	1.858	(1.139–3.031)	0.005	2.044	(1.236–3.382)	0.007	2.071	(1.225–3.501)	0.014	1.944	(1.144–3.302)

*Model I: a univariate model without controlling for any confounding factors. Model II: controls for maternal demographic characteristics (maternal age, ethnicity, education and occupation). Model III: based on model II supplemented to control for demographic characteristics of the spouse (age, ethnicity, education and occupation). Model IV: based on model III, supplemented to control for pre-pregnancy BMI, primipara, history of abnormal pregnancy, history of cesarean section*.

a *: based on model IV, supplemented to control for anemia, vaginitis, infection*.

### Factors Affecting Adverse Pregnancy Outcomes During the COVID-19 Pandemic

We analyzed the factors related to adverse pregnancy outcomes in 172 women in the pandemic group and 139 women in the pre-pandemic group. In a univariate analysis, the rate of stay-at-home was significantly higher in pregnant women (20.47 vs. 10.95%, *P* = 0.024) and their spouses (19.63 vs. 10.45%, *P* = 0.029) in the pandemic group, and higher risk of anemia (47.67 vs. 35.97%, *P* = 0.038) and vaginal candidiasis (5.20 vs. 0.72%, *P* = 0.055) in the pandemic group. However, compared with the pre-pandemic group, women in the pandemic group had a lower rate of exposure to smoking (14.91 vs. 26.67%, *P* = 0.027), tea drinking (4.39 vs. 15.83%, *P* = 0.004) in the first trimester. Moreover, women in the pandemic group had fewer exercises, such as reduced average walking time per week (4.65 vs. 5.69 h), and reduced proportion of participating in physical activities except walking (at least three times per week, at least 30 min each time) (12.5 vs. 18.33%), but it was not statistically significant (*P* = 0.22) (Table 4).

**Table 4:** Univariate analysis of adverse pregnancy outcomes during pandemic, China, 2018-2020.

		**Pregnant women with adverse pregnancy outcomes, No./total No**.					
		**Pandemic (*n* = 172)**	**%**	**Pre-pandemic group (*n* = 139)**	**%**	**X^2^/Z**	***P*-value**
**Sociodemographic**	**Maternal age (years)**	32.91 ± 4.04		32.71 ± 4.13		−0.47	0.641
	**Maternal occupation**					5.07	0.024^*^
	Employed	136 / 171	79.5	122 / 137	89.1		
	Stay-at-home	35 / 171	20.5	15 / 137	10.9		
	**Spouse occupation**					4.74	0.029 ^*^
	Employed	131 / 163	80.4	120 / 134	89.6		
	Stay-at-home	32 / 163	19.6	14 / 134	10.4		
	**Maternal education level**						0.839
	Junior college degree or below	98 / 172	57.0	76 / 139	54.7	0.17	0.685
	Bachelor degree or above	74 / 172	43.0	63 / 139	45.3		
	**Spouse education level**						0.318
	Junior college degree or below	89 / 170	52.4	82 / 138	59.4	1.54	0.215
	Bachelor degree or above	81 / 170	47.6	56 / 138	40.6		
**Medical**	**Pre-pregnancy BMI (kg/m** ^**2**^ **)**	23.27 ± 4.27		23.08 ± 3.97		−0.08	0.935
	**Primipara**					2.298	0.13
	yes	41 / 167	24.6	45 / 139	32.4		
	no	126/ 167	29.3	94 / 139	31.7		
	History of abnormal pregnancy	8 / 172	4.7	5 / 139	3.6	0.21	0.644
	History of cesarean section	31 / 172	18.0	25 / 139	18.0	0.00	0.993
	Anemia	82 / 172	47.7	50 / 139	36.0	4.31	0.038 ^*^
	Vaginal candidiasis	9 / 172	5.20	1 / 139	0.72	3.69	0.055
**Life style^a^**	Smoking	4/114	3.51	3/120	2.50	0.01	0.945
	Exposure to smoking	17 / 114	14.9	32 / 120	26.7	4.88	0.027 ^*^
	Alcohol drinking	3/114	2.6	1/120	0.8	0.31	0.578
	Tea drinking	5 / 114	4.4	19 / 120	15.8	8.32	0.004 ^*^
	Nutrient supplement^b^	68 / 112	60.7	69 / 120	57.5	0.25	0.619
	Using computers(h/day)	3.02 ± 3.43		3.71 ± 3.32		−1.93	0.053
	Watching TV(h/day)	1.77 ± 2.15		1.95 ± 1.55		−2.20	0.028 ^*^
	**Physical activities^c^**					1.50	0.220
	yes	14 / 112	12.5	22 / 119	18.3		
	no	98 / 112	87.5	97 / 119	80.8		
	Walking (h/week)	4.65 ± 5.95		5.69 ± 7.19		−0.86	0.390

* *P < 0.05*.

a *: data derived from first trimester*.

b *: Nutrient supplement: refers to vitamin or mineral supplements (e.g., folic acid, calcium tablets, iron, etc)*.

c *: Physical activities: Min at least 3 times a week for 30 min each, except walking*.

Furthermore, we conducted a multivariate analysis using a binary logistic regression model to predict the risk factors affecting adverse pregnancy outcomes during the pandemic. Compared with the pre-pandemic group, women who had adverse pregnancy outcomes in the pandemic group often had anemia (OR = 2.002, CI = 1.080~3.708, *P* = 0.027), less exposure to smoking (OR = 0.456 CI = 0.213~0.974, *P* = 0.043) and less tea drinking (OR = 0.193 CI = 0.057–0.653, *P* = 0.008) during pregnancy (Table 5).

**Table 5:** Hierarchical logistic regression predicting adverse pregnancy outcomes from sociodemographic features, medical records and life behaviors, China, 2018-2020.

	**Step 1**			**Step 2**			**Step 3**		
	***P*-value**	**OR**	**95% CI**	***P*-value**	**OR**	**95% CI**	***P*-value**	**OR**	**95% CI**
Maternal occupation	0.178	0.614	0.303–1.248	0.232	0.642	0.311–1.327	0.639	0.802	0.319–2.017
Spouse occupation	0.089	0.529	0.254–1.102	0.092	0.523	0.246–1.112	0.115	0.48	0.193–1.194
Maternal education level	0.395	0.783	0.445–1.376	0.55	0.838	0.471–1.493	0.674	0.849	0.396–1.82
Spouse education level	0.1	1.609	0.912–2.838	0.082	1.675	0.936–3	0.24	1.543	0.749–3.181
Maternal age	0.843	0.994	0.938–1.053	0.459	0.976	0.915–1.041	0.215	0.95	0.877–1.03
Pre-pregnancy BMI				0.839	1.006	0.948–1.068	0.23	1.045	0.972–1.124
Primipara				0.053	0.556	0.306–1.008	0.246	0.634	0.293–1.37
History of abnormal pregnancy				0.864	0.901	0.272–2.985	0.41	0.562	0.143–2.209
History of cesarean section				0.506	0.798	0.409–1.554	0.629	1.22	0.544–2.737
Anemia				0.051	1.629	0.998–2.659	0.027^*^	2.002	1.08–3.708
Exposure to smoking							0.043^*^	0.456	0.213–0.974
Tea drinking							0.008^*^	0.193	0.057–0.653
Nutrient supplement							0.258	1.423	0.772–2.622
Using computers, h/day							0.245	0.945	0.858–1.04
Watching TV, h/day							0.992	1.001	0.85–1.178
Physical activities							0.19	0.568	0.243–1.325
Walking, h/week							0.319	0.975	0.928–1.025

* *P < 0.05*.

## Discussion

In this study, we systematically analyzed the impact of COVID-19 pandemic on the pregnancy outcomes of uninfected pregnant women from the Shunyi District of Beijing and explored the medical, social and environmental factors associated with adverse pregnancy outcomes during the pandemic. Our research showed that more pregnant women were non-primiparous and an increased proportion of pregnant women's spouses stayed at home during the COVID-19 pandemic. Anemia, vaginitis (especially vaginal candidiasis), adverse pregnancy outcomes (especially preterm birth), shoulder dystocia were higher, but postpartum hemorrhage was lower in the pandemic group than that in the pre-pandemic group. In addition, the proportion of adverse pregnancy outcomes increased during the pandemic period, which was correlated to maternal anemia and vaginal candidiasis.

We observed a significant increase in the incidence of shoulder dystocia during the COVID-19 pandemic that has not been reported in other studies. It has been suggested that shoulder dystocia is related to fetal weight, especially larger-gestational age infants and macrosomia (20–22). In line with previous studies, the incidence of macrosomia and larger-gestational age infants in the pandemic group was also higher in our cohort. Physical activity during pregnancy can prevent excessive fetal growth and reduce the incidence of macrosomia (23). We found less exercise and physical activity in pregnant women during the pandemic, which may affect the incidence of macrosomia and shoulder dystocia.

In our study, we found that an increase in adverse pregnancy outcomes during the pandemic was related to vaginal candidiasis in pregnant women. Preterm birth was one of the significantly increased adverse outcomes in our analysis, which was consistent with previous studies conducted during the COVID-19 pandemic (18, 24). Preterm birth could be caused by a variety of mechanisms including infection or inflammation, stress, and other immune-mediated processes (25, 26). It is possible that *Candida* colonization disrupts the normal vaginal flora, resulting in a decrease in *Lactobacillus* species and an increase in the number of pro-inflammatory microorganisms. Microorganisms may spread from the vagina to the uterus via upward infection to increase the risk of preterm delivery (25, 27).

Moreover, the increases in adverse outcomes may partially be related to limited access to health care for women's newborns during the COVID-19 pandemic. Lockdowns such as home isolation and restrictions on public activities, and reductions in hospital staff may affect the routine checkups during pregnancy and lead to insufficient health care guidance (17, 28, 29). A study has reported that women did not have enough antenatal visits due to lockdown and fear of infection, resulting in 44.70% of pregnant women suffering from one or more complications (30). Given the government guidance and the personal safety concerns during the pandemic, telemedicine maybe a preferable choice which enables medical intervention to continue. For example, telemedicine together with home monitoring of blood glucose and blood pressure were used at a hospital in Nanjing, China (31), which reduced the hospital visits from 500 to 200 per week with no known associated change in pregnancy outcomes.

Our research used a nested case-control design based on the CHALLENGE study which covered pre-pandemic and pandemic study period. To explore the possible factors associated with the increased risk of adverse pregnancy outcomes during the pandemic, we utilized a comprehensive dataset collected from multiple systems in the hospital (e.g., medical record system, inspection system, and B-ultrasound system, etc.) by integrating the demographic and medical records of the participants, as well as the questionnaire on physical exercise and psychological conditions. Shunyi Maternal and Children's Hospital of Beijing Children's Hospital is responsible for the medical care of women and children in the district, and the enrolls in the cohort are people who stably live in Shunyi, therefore the long-term impact of the pandemic on pregnant women and newborns are possible in the follow-up studies. However, our study is a single-center study that focused on an area of Beijing. A more comprehensive analysis of the maternal and infant health of uninfected pregnant women during the pandemic can be conducted in future in a multi-center investigation to provide nationwide information.

Our results showed that pregnant women had an increased risk of adverse pregnancy outcomes, particularly preterm birth, during the COVID-19 pandemic. The proportion of adverse pregnancy outcomes was increased, which was related to maternal anemia and vaginal candidiasis. In addition, the proportion of shoulder dystocia, vaginitis, and anemia in pregnant women was higher than that in the pre-pandemic group. Our findings emphasize the significance of regular prenatal checkups and a healthy lifestyle in lowering the risk of adverse pregnancy outcomes. Meanwhile, our findings highlight the importance of developing timely strategies to ensure earlier diagnose and intervention of high-risk pregnant women.

## Data Availability Statement

The raw data supporting the conclusions of this article will be made available by the authors, without undue reservation.

## Ethics Statement

The studies involving human participants were reviewed and approved by Shunyi Maternal and Children's Hospital of Beijing Children's Hospital. The patients/participants provided their written informed consent to participate in this study.

## Author Contributions

WL designed the research, coordinated the project, acquisition the funding, and revised the manuscript. XN coordinated the project and supervised the study. XL verified the statistical analyses and wrote the draft. ChoH performed the statistical analyses and wrote the draft. FJ collected and analyzed the data. ChaH coordinated the project and participated in the design and interpretation of the data. XZ led the questionnaires. LX managed the cohort. YZ designed the cohort. All authors contributed to the article and approved the submitted version.

## Funding

This work was supported by a grant from the Ministry of Science and Technology of China (#2016YFC1000306).

## Conflict of Interest

The authors declare that the research was conducted in the absence of any commercial or financial relationships that could be construed as a potential conflict of interest.

## Publisher's Note

All claims expressed in this article are solely those of the authors and do not necessarily represent those of their affiliated organizations, or those of the publisher, the editors and the reviewers. Any product that may be evaluated in this article, or claim that may be made by its manufacturer, is not guaranteed or endorsed by the publisher.
