# Supplementary material for: Evaluation of the Effects on Uninfected Pregnant Women and Their Pregnancy Outcomes During the COVID-19 Pandemic in Beijing, China
Source: Front Med (Lausanne). 2022 May 11;9:842826. doi: 10.3389/fmed.2022.842826 (PMC9131041; doi:10.3389/fmed.2022.842826)
Supplement: Supplementary file 1 [file Table_1.docx]

**Questionnaire (baseline)**

1. Your name?
2. Your ID number: |__|__|__|__|__|__|__|__|__|__|__|__|__|__|__|__|__|__|
3. Your date of birth: (Nong / Yang) calendar ________ year _____ month _____
4. Your education level:

1. No formal education 2. Primary school 3. Junior high school 4. High school, secondary school 5. College 6. University 7. Master's degree or above 9. Unknown

1. Your home address?
2. Your work status? Address?
3. Name of the child's father: ______________________
4. His ID number: |__|__|__|__|__|__|__|__|__|__|__|__|__|__|__|__|__|__|
5. His date of birth: (Nong / Yang) Calendar ________ year _____ month _____
6. His nationality: _________
7. His specific job content is _____________
8. His education level:

1. No formal education 2. Primary school 3. Junior high school 4. High school, secondary school 5. College 6. University 7. Master's degree or above 9. Unknown

1. His work status? Address?
2. Your weight at birth is: _______kg
3. Did you grow up breastfeeding yourself?

0. No 1. Yes 9. Not sure

1. What was your weight before pregnancy: ___________kg
2. What is your current weight: _____________kg
3. What is your current height: _____________cm
4. What date was the last menstrual period?
5. How many times have you been pregnant before? (excluding this pregnancy)
6. Have you been diagnosed as any type of diseases before?

**Questionnaire (12-13 week)**

Living Behaviors and Environmental Exposures

1. Have you ever smoked regularly (at least one per day for more than 1 month)?

0. No 1. Yes_______

1. Have you ever been exposed to smoking since this pregnancy?

0. No 1. Yes_______

1. Have you ever been drinking alcohol? (meaning at least once a week on average)

0. No 1. Yes_______

1. Do you ever drink tea often? (meaning at least 3 times a week)

0. No 1. Yes________

1. During the 1 year before this pregnancy, did you regularly take vitamin or mineral supplements? (Such as folic acid, calcium tablets, iron, etc.)

0. No 1. Yes________

1. Have you taken vitamin or mineral supplements since this pregnancy?

0. No 1. Yes________

1. Since pregnancy, you have walked an average of ________ hours per week.
2. Have you participated in physical activity (except walking, at least 3 times a week for at least 30 minutes) since pregnancy?

0. No 1. Yes________

1. Since pregnancy, you have used the computer _______ hours per day on average.
2. Since pregnancy, on average, you watch _______ hours of TV per day.
